# Roles of FGFs as Adipokines in Adipose Tissue Development, Remodeling, and Metabolism

**DOI:** 10.3389/fendo.2014.00018

**Published:** 2014-02-24

**Authors:** Hiroya Ohta, Nobuyuki Itoh

**Affiliations:** ^1^Department of Genetic Biochemistry, Kyoto University Graduate School of Pharmaceutical Sciences, Kyoto, Japan

**Keywords:** adipocyte, adipokine, development, FGF, metabolism, remodeling

## Abstract

White and brown adipose tissues (BATs), which store and burn lipids, respectively, play critical roles in energy homeostasis. Fibroblast growth factors (FGFs) are signaling proteins with diverse functions in development, metabolism, and neural function. Among 22 FGFs, FGF1, FGF10, and FGF21 play roles as adipokines, adipocyte-secreted proteins, in the development and function of white and BATs. FGF1 is a critical transducer in white adipose tissue (WAT) remodeling. The peroxisome proliferator-activated receptor γ–FGF1 axis is critical for energy homeostasis. FGF10 is essential for embryonic white adipocyte development. FGF21 activates BAT in response to cold exposure. FGF21 also stimulates the accumulation of brown-like cells in WAT during cold exposure and is an upstream effector of adiponectin, which controls systemic energy metabolism. These findings provide new insights into the roles of FGF signaling in white and BATs and potential therapeutic strategies for metabolic disorders.

## Introduction

White adipose tissue (WAT), a lipid storage site, plays a critical role in energy homeostasis. Obesity, the excessive development of WAT, is a well-known risk factor for several diseases including diabetes, hypertension, and atherosclerosis (1, 2). Brown adipose tissue (BAT) burns lipids and dissipates chemical energy as protection against hypothermia and obesity (3). Therefore, understanding the molecular and cellular mechanisms of adipose tissue development and metabolism has become a priority. WAT is also a dynamic tissue that actively communicates by sending adipocyte-secreted proteins, adipokines, which act in an autocrine/paracrine or endocrine manner. This secretory function has been highlighted in relation to adipose tissue development, remodeling, and metabolism (1, 2). Although BAT also produces adipokines, the endocrine roles of BAT are needed to determine by further research (4).

Fibroblast growth factors (FGFs) are signaling proteins with diverse functions in development, metabolism, and neural function. The FGF family comprises 22 members (5). Among these FGFs, FGF1, FGF10, and FGF21 have been shown to play roles as adipokines in WAT development, remodeling, and metabolism. FGF21 also activates BAT as an adipokine. These findings provide new insights into their roles in adipose tissues and provide potential therapeutic strategies for obesity and metabolic disorders. A succinct review of the roles of FGFs as adipokines is provided in this article.

## The FGF Family

**Figure 1:**
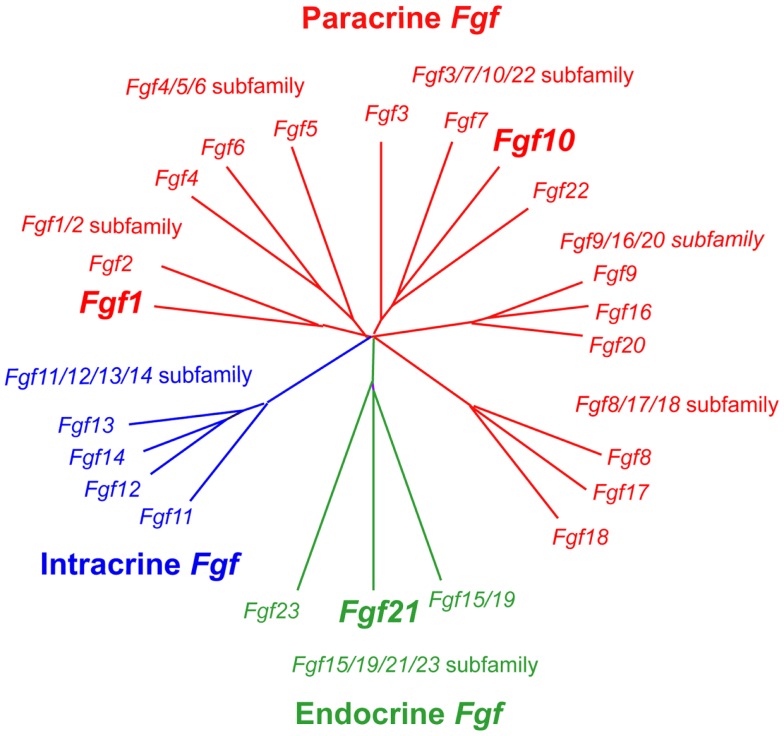
**Evolutionary relationships within the *Fgf* gene family by phylogenetic analysis**. Phylogenetic analysis shows that 22 *Fgf* genes can be arranged into seven subfamilies containing two to four members each. Branch lengths are proportional to the evolutionary distance between each gene (5).

Fibroblast growth factors are proteins with ~150–300 amino acids and a conserved core of ~120 amino acids (~30–60% identity). Phylogenetic analysis of the *Fgf* gene family identifies seven subfamilies, indicating potential evolutionary relationships in this gene family. FGFs are also classified as intracrine, paracrine, and endocrine FGFs by their action mechanisms (Figure 1). Intracrine FGFs, which are not secreted extracellularly, play roles in the regulation of electrical excitability in neurons in an intracrine manner. Paracrine FGFs, secreted FGFs, mediate biological responses by binding to cell surface FGF receptors (FGFRs) with heparin/heparan sulfate as a cofactor, which is necessary for stable interactions with FGFRs and local signaling (5). Seven major FGFR proteins (FGFRs 1b, 1c, 2b, 2c, 3b, 3c, and 4) with differing ligand-binding specificity are generated from *Fgfr1, Fgfr2, Fgfr3*, and *Fgfr4* genes by alternative splicing. FGF binding to FGFRs induces the activation of four key intracellular signaling pathways: RAS–RAF–MAPK, PI3K–AKT, STAT, and PLCγ (6). Endocrine FGFs also mediate their biological responses in an FGFR-dependent manner. However, they do not require heparin/heparan sulfate, which enables endocrine FGFs to function in an endocrine manner. α- and β-Klotho, single-pass transmembrane proteins with short cytoplasmic domains, are essential for endocrine FGF signaling as cofactors (5, 7).

## Adipokines

White adipose tissue mainly comprises adipocytes. Preadipocytes, adipocyte precursors cells, are also present in WAT. Adipogenesis is the differentiation of preadipocytes to adipocytes. Expansion of the adipose tissue mass is caused by a combination of size increases in the pre-existing adipocytes and adipogenesis. Some adipokines, adipocyte-secreted proteins including leptin and adiponectin play roles in the regulation of appetite, food intake, and energy homeostasis in an endocrine manner. Other adipokines including tumor necrosis factor α and interleukin-6 play roles in adipose tissue remodeling, adipogenesis, and angiogenesis in an autocrine/paracrine manner (1, 2). Secreted adipokines including insulin-like growth factor 1 and interleukin-6 are produced in BAT in response to cold exposure (4). FGF1, FGF10, and FGF21 also play roles as autocrine/paracrine adipokines in WAT or BAT.

## FGF1

FGF1 is not a typical secreted protein and may be released from damaged cells or in an exocytotic mechanism. FGF1 mediates biological responses by activating FGFRs. However, *Fgf1* knockout mice normally have no significant phenotype (5).

*Fgf1* is highly induced in WAT by a high-fat diet. The nuclear receptor peroxisome proliferator-activated receptor γ (PPARγ) is the adipocyte master regulator and target of the thiazolidinedione class of insulin sensitizing drugs (8). FGF1 induction is regulated by PPARγ. *Fgf1* knockout mice develop an aggressive diabetic phenotype coupled to aberrant adipose expansion by a high-fat diet. In addition, they show multiple histopathologies in the vasculature network, an accentuated inflammatory response, and aberrant adipocyte size distribution. However, this inflamed adipose tissue fails to resolve properly, resulting in extensive white fat necrosis with the withdrawal of the high-fat diet (9). WAT remodeling in nutrient availability is essential to maintain metabolic homeostasis (10). These findings indicate that FGF1 is a critical transducer in WAT remodeling and that the PPARγ–FGF1 axis is critical for maintaining metabolic homeostasis and insulin sensitization. Although which FGFR mediates the FGF1 action remains unclear, the axis provides the therapeutic potential of FGF1 in potentially mediating insulin sensitization (Table S1 in Supplementary Material).

## FGF10

**Figure 2:**
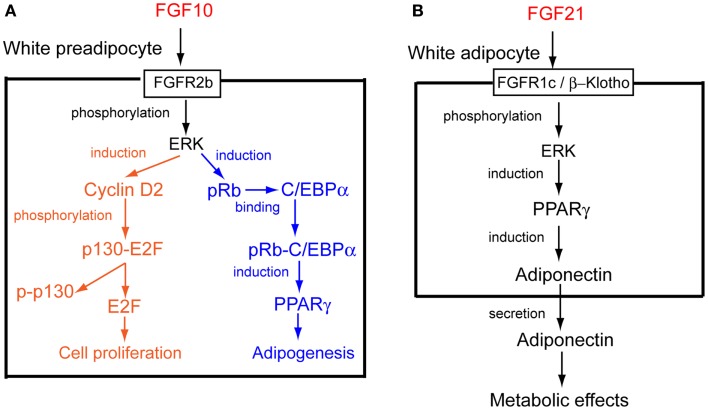
**Action mechanisms of FGF10 and FGF21**. **(A)** FGF10 acts on white preadipocytes in an autocrine/paracrine manner. The possible mechanisms of FGF10-induced cell proliferation and adipogenesis in white preadipocytes are shown (14). **(B)** FGF21 acts on white adipocytes in an autocrine/paracrine manner. The possible mechanism of FGF21-induced adiponectin production in white adipocytes is shown (25).

FGF10 is a paracrine FGF, for which FGFR2b is a specific receptor. *Fgf10* knockout mice die shortly after birth with impaired multi-organ formation, which indicates that FGF10 is critical for multi-organ formation (5). Thus, its roles at postnatal stages remain unclear. *Fgf10* is abundantly expressed in WAT, in which *Fgf10* is particularly expressed by preadipocytes. WAT development with markedly less proliferative activity is greatly impaired in *Fgf10* knockout mouse embryos. The Ras/MAPK pathway, activated through FGFR2b by FGF10, is essential for its mitogenic activity in preadipocytes (Figure 2A) (11, 12).

The retinoblastoma family proteins, pRb and p130, are involved in the cell cycles of various cells. They bind to E2Fs in quiescent cells, leading to the repression of target genes involved in the cell cycle. When quiescent cells are stimulated to enter the cell cycle, retinoblastoma family proteins are subjected to cyclin-dependent phosphorylation to release E2Fs, which advance the cell cycle (13). *Cyclin D2* expression and p130 phosphorylation are impaired in the WAT of *Fgf10* knockout mouse embryos. FGF10 stimulates *cyclin D2* expression and p130 phosphorylation in cultured cells. Thus, FGF10 stimulates cell proliferation through the activation of FGFR2b and Ras/MAPK pathway, followed by the cyclin D2-dependent phosphorylation of p130 in WAT (Figure 2A) (Table S1 in Supplementary Material) (14).

Adipogenesis is the process by which preadipocytes differentiate into mature adipocytes. The CCAAT/enhancer binding protein α (C/EBPα) and PPARγ are required for adipogenesis. Although *Pparγ* expression is markedly decreased in the WAT of *Fgf10* knockout mouse embryos, *C/ebpα* expression is essentially unchanged. *Pparγ* expression is markedly reduced in the WAT of *C/ebpα* knockout mice. However, *Fgf10* expression is essentially unchanged. In addition, *Fgf10* and *C/ebpα* expression in the WAT of wild-type embryos is followed by *Pparγ* expression (12). The number of pRb-positive cells is markedly decreased in the WAT of *Fgf10* knockout mouse embryos, although pRb phosphorylation is not inhibited (14). *pRb* knockout fibroblasts do not differentiate into adipocytes. In addition, pRb binds directly to C/EBPs and stimulates the activity of C/EBPs (15). Thus, FGF10 induces pRb production by activating FGFR2b and the Ras/MAPK pathway. pRb binds C/EBPα and induces the expression of *Pparγ*, followed by the stimulation of adipogenesis (Figure 2A) (Table S1 in Supplementary Material) (14).

## FGF21

FGF21 mainly acts as a hepatic endocrine regulator, a hepatokine, in glucose and lipid metabolism. Hepatic *Fgf21* expression is markedly induced in mice by fasting or a ketogenic diet. The results from experiments using *Fgf21* transgenic mice and cultured cells demonstrate that FGF21 exerts pharmacological effects on glucose and lipid metabolism in hepatocytes and white adipocytes via cell surface FGFRs. *Fgf21* transgenic mice are resistant to diet-induced obesity. Serum glucose levels are also reduced to near normal levels in both *ob/ob* and *db/db* mice by the administration of FGF21. These findings indicate that FGF21 plays a role in glucose metabolism and has potential therapeutic effects on metabolic diseases. However, the results from experiments using *Fgf21* knockout mice reveal that FGF21 inhibits lipolysis in white adipocytes during fasting and attenuates torpor induced by a ketogenic diet, but may be not a physiological regulator for these hepatic functions. These findings suggest that its pharmacological effects are distinct from its physiological roles (16, 17).

Brown adipose tissue expresses FGFR1c and β-Klotho, a strong candidate receptor and co-receptor for FGF21 signaling, respectively. Exogenous FGF21 activates BAT (18). Cold exposure also activates β-adrenergic receptors on brown adipocytes. This process induces mitochondrial uncoupling protein 1 (UCP1), which releases chemical energy as heat by uncoupling oxidative phosphorylation. The induction of co-activator PGC-1α by cold exposure induces *Ucp1* expression (3). In addition to secreted adipokines including insulin-like growth factor 1 and interleukin-6, which are produced in BAT in response to cold exposure (4), FGF21 is also synthesized in BAT in response to cold exposure. These findings indicate that FGF21 activates BAT as an adipokine in an autocrine/paracrine manner (Table S1 in Supplementary Material) (19).

In addition to BAT, UCP1-positive, brown fat-like cells can emerge in WAT with prolonged cold exposure (20). The marked accumulation of brown-like cells can be found most readily in subcutaneous WAT. This process is characterized by the appearance of UCP1-positive, multilocular adipocytes. WAT also expresses *Fgf21, Fgfr1c*, and β*-Klotho* (18). *Fgf21* knockout mice display an impaired ability to adapt to chronic cold exposure, with the diminished browning of WAT. FGF21 produced in WAT increases the expression of *Ucp1* and other thermogenic genes in an autocrine/paracrine manner. FGF21 regulates this process, at least in part, by enhancing adipose tissue PGC-1α protein levels. These findings indicate that FGF21 activates the thermogenic machinery to provide a robust defense against hypothermia (Table S1 in Supplementary Material) (21).

In WAT, FGF21, which forms a feed-forward loop with PPARγ, mediates the metabolic benefits of PPARγ on glucose homeostasis and insulin sensitivity in an autocrine/paracrine manner (22). In lipodystrophic mice with less WAT, systemic FGF21 administration is not effective in decreasing blood glucose levels or increasing insulin sensitivity. However, FGF21 is effective after the transplantation of WAT into these mice (23). These findings indicate that WAT is a predominant site conferring the antidiabetic activities of FGF21.

Adiponectin has many functional similarities to FGF21. Adiponectin as an adipokine controls systemic glucose and lipid homeostasis in the liver and skeletal muscle in an endocrine manner. Furthermore, adiponectin is a downstream effector of PPARγ and an essential mediator for many therapeutic benefits of the PPARγ agonists TZDs, including insulin sensitization and vascular protection (24). FGF21 enhances both the expression and secretion of adiponectin in white adipocytes and serum levels of adiponectin in mice. *Adiponectin* knockout mice are refractory to several therapeutic benefits of FGF21. Furthermore, the effects of FGF21 on the attenuation of obesity-induced impairments in insulin signaling in the liver and skeletal muscle are abrogated in *adiponectin* knockout mice. However, the FGF21-mediated activation of ERK1/ERK2 in WAT remains unaffected in *adiponectin* knockout mice. These findings indicate that adiponectin couples FGF21 actions as a downstream effector of FGF21 in white adipocytes and mediates the systemic effects of FGF21 on energy metabolism and insulin sensitivity in the liver and skeletal muscle (Figure 2B) (Table S1 in Supplementary Material) (25, 26).

Insulin resistance arises from the aberrant accumulation of intracellular lipids, including the sphingolipid ceramide, in insulin-responsive tissues. FGF21 stimulates adiponectin secretion and diminishes the accumulation of ceramides in obese animals. *Adiponectin* knockout mice are refractory to changes in energy expenditure and the ceramide-lowering effects evoked by FGF21 administration (26). These findings indicate that an FGF21–adiponectin–ceramide axis controls energy expenditure and insulin action.

## Conclusion

Although WAT and BAT store and burn lipids, respectively, they are also dynamic tissues that actively communicates by sending different types of adipokines, which mainly play roles in energy homeostasis. FGFs are signaling proteins with diverse functions in development, metabolism, and neural function. In addition, FGF1, FGF10, and FGF21 have been shown to be adipokines with crucial roles in WAT or BAT functions, suggesting new roles for FGFs and potential therapeutic strategies for metabolic disorders.

## Conflict of Interest Statement

The authors declare that the research was conducted in the absence of any commercial or financial relationships that could be construed as a potential conflict of interest.

## Supplementary Material

The Supplementary Material for this article can be found online at http://www.frontiersin.org/Journal/10.3389/fendo.2014.00018/abstract
