# Supplementary material for: Roles of FGFs as Adipokines in Adipose Tissue Development, Remodeling, and Metabolism
Source: Front Endocrinol (Lausanne). 2014 Feb 24;5:18. doi: 10.3389/fendo.2014.00018 (PMC3932445; doi:10.3389/fendo.2014.00018)
Supplement: Supplementary file 1 [file 76669_Itoh_DataSheet1.DOC]

**Table 1 I Roles of FGFs 1, 10, and 21 as autocrine/paracrine adipokines**

**in adipose tissue development, remodeling, and metabolism.**

**Production /action site** **Role** **FGFR / Cofactor**

**FGF1**  White adipocytes Adipose tissue remodeling FGFR / Heparan sulfate

and metabolism

**FGF10** White preadipocytes Preadipocyte development FGFR2b / Heparan sulfate

**FGF21** Brown adipocytes Adipocyte activation FGFR1c / β-Klotho

White adipocytes Adipocyte browning FGFR1c / β-Klotho

and adiponectin production
